# Distributed Representations Accelerate Evolution of Adaptive Behaviours

**DOI:** 10.1371/journal.pcbi.0030147

**Published:** 2007-08-03

**Authors:** James V Stone

**Affiliations:** Psychology Department, Sheffield University, Sheffield, United Kingdom; University College London, United Kingdom

## Abstract

Animals with rudimentary innate abilities require substantial learning to transform those abilities into useful skills, where a skill can be considered as a set of sensory–motor associations. Using linear neural network models, it is proved that if skills are stored as distributed representations, then within-lifetime learning of part of a skill can induce automatic learning of the remaining parts of that skill. More importantly, it is shown that this “free-lunch” learning (FLL) is responsible for accelerated evolution of skills, when compared with networks which either 1) cannot benefit from FLL or 2) cannot learn. Specifically, it is shown that FLL accelerates the appearance of adaptive behaviour, both in its innate form and as FLL-induced behaviour, and that FLL can accelerate the rate at which learned behaviours become innate.

## Author Summary

Some behaviours are purely innate (e.g., blinking), whereas other, “apparently innate,” behaviours require a degree of learning to refine them into a useful skill (e.g., nest building). In terms of biological fitness, it matters how quickly such learning occurs, because time spent learning is time spent not eating, or time spent being eaten, both of which reduce fitness. Using artificial neural networks as model organisms, it is proven that it is possible for an organism to be born with a set of “primed” connections which guarantee that learning part of a skill induces automatic learning of other skill components, an effect known as free-lunch learning (FLL). Critically, this effect depends on the assumption that associations are stored as distributed representations. Using a genetic algorithm, it is shown that primed organisms can evolve within 30 generations. This has three important consequences. First, primed organisms learn quickly, which increases their fitness. Second, the presence of FLL effectively accelerates the rate of evolution, for both learned and innate skill components. Third, FLL can accelerate the rate at which learned behaviours become innate. These findings suggest that species may depend on the presence of distributed representations to ensure rapid evolution of adaptive behaviours.

## Introduction

Both evolution and learning may be considered as different types of adaptation. Learning occurs within a lifetime, whereas genetic change occurs across lifetimes [1]. Whereas genetic change ensures that a task can be executed innately, learning permits even the most rudimentary innate ability to be honed into a useful skill.

In an environment that fluctuates from generation to generation, learning permits an innate ability to be adapted to the particular physical environment into which each generation is born. If the environment ceases to fluctuate, then genetic assimilation [2] can transform a rudimentary innate ability, which requires much learning, into an innate skill, which requires minimal learning. This transformation is more likely to occur if the cost of learning is high [3,4], and, in this case, computer simulations suggest that learning can accelerate the rate of genetic assimilation [5] via the Baldwin effect [6]. However, if learning is sufficiently inexpensive, then genetic change may not occur at all [7,8]. Overall, there appears to be a tradeoff between learning and genetic assimilation, such that learning can subsidize genetic change, especially if learning is inexpensive.

All but the most primitive organisms learn in order to survive, and organisms which learn quickly are at a selective advantage relative to those that learn slowly. Therefore, a mechanism which reduces the time required to learn a given behaviour confers a selective advantage. One candidate for such a mechanism is FLL [9,10].

As explained below, FLL ensures that in the process of learning one set of associations or behaviours another set of associations is usually learned. These associations could comprise either perceptual skills (such as face recognition, predator recognition [11], or prey recognition), or motor skills (such as catching prey, flying, seed pecking, or nest building).

### Free-Lunch Learning

Before considering how FLL can accelerate evolution of certain types of behaviours, FLL will be described in its original context of spontaneous recovery of memory in humans [9] and in neural network models [10]. Note that FLL is not unique to a specific class of network architectures, although it does assume that associations are learned using a form of supervised learning.

In humans, FLL has been demonstrated using a task in which participants learned the positions of letters on a nonstandard computer keyboard [9]. After a period of forgetting, participants relearned a proportion of these letter positions. Crucially, it was found that this relearning induced recovery of the non-relearned letter positions.

More recently, a set of theorems provided a formal characterization of FLL in linear neural network models [10]. In essence, FLL occurs in neural network models because each association is distributed amongst all connection weights (synapses) between units (model neurons). After partial forgetting, relearning some of the associations forces all of the weights closer to pre-forgetting values, resulting in improved performance even on non-relearned associations; a general proof is provided in [10]. A geometric demonstration of FLL for a network with two connection weights is given in Figure 1. Networks with multiple input and output units can be considered without loss of generality [10].

**Figure 1:**
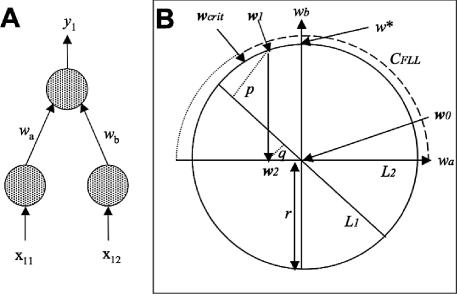
Geometry of Free-Lunch Learning (A) Given a network with two input units and one output unit, its connection weights *w_a_* and *w_b_* define a weight vector **w** = (*w_a_*,*w_b_*). The network learns two subsets *A*_1_ and *A*_2_. In this example, each subset is a single association, where *A*_1_ (for example) is the mapping from input vector **x**_1_ = (*x*_11_,*x*_12_) to desired output value *d*_1_, and learning *A*_1_ consists of adjusting **w** until the network output *y*_1_ = **w** · **x**_1_ equals *d*_1_. (B) Each association *A*_1_ and *A*_2_ defines a constraint line *L*_1_ and *L*_2_, respectively. The intersection of *L*_1_ and *L*_1_ defines a point **w**_0_ which satisfies both constraints, so that zero performance error on *A* = *A*_1_ ∪ *A*_2_ is obtained if **w** = **w**_0_. After partial forgetting, the weight vector is a randomly chosen point **w**_1_ on the circle *C* of radius *r*, and the performance error on *A*_1_ is given by the squared distance *p* ^2^. After relearning *A*_2_, the weight vector **w**_2_ lies in *L*_2_, and performance error on *A*_1_ is the squared distance *q* ^2^. FLL occurs if performance error on *A*_1_ is decreased by relearning *A*_2_, or equivalently if *p* ^2^ > *q* ^2^. Relearning *A*_2_ has three possible effects, depending on the position of **w**_1_ on *C*: 1) if **w**_1_ is under the larger (dashed) arc *C_FLL_* (as shown here), then *p* ^2^ > *q* ^2^; therefore, FLL is observed, 2) if **w**_1_ is under the smaller (dotted) arc, then *p* ^2^ < *q* ^2^; therefore, negative FLL is observed, and 3) if **w**_1_ is at the critical point **w** *_crit_*, then *p* ^2^ = *q* ^2^; therefore, no FLL is observed. Given that **w**_1_ is a randomly chosen point on the circle *C*, the probability of FLL is equal to the proportion of the upper semicircle under the (dashed) arc *C_FLL_*. Symmetry implies the above analysis also applies to the lower half of *C*. In terms of evolution, a network “born” with **w** = **w** ^*^ has zero error on both *A*_1_ and *A*_2_ after learning only *A*_2_ (see text).

The protocol used to examine FLL in neural networks is as follows (see Figure 2). A network with *n* input units and one output unit has *n* connection weights. This network learns a set *A* of *m* ≤ *n* associations, where *A* = *A*_1_ ∪ *A*_2_ comprises two subsets *A*_1_ and *A*_2_ of *n*_1_ and *n*_2_ associations, respectively (note that *m* = *n*_1_ + *n*_2_). After the associations *A* have been learned and then partially forgotten, performance error on subset *A*_1_ is measured (forgetting is induced by adding isotropic noise to weights). Finally, *only A*_2_ is relearned, and then performance error on *A*_1_ is remeasured. FLL occurs if relearning *A*_2_ improves performance on *A*_1_. It has been proven that the probability of FLL approaches unity as the number of weights increases [10]. For the sake of brevity, this is reflected in phrases such as “learning *A*_2_ *usually* improves performance on *A*_2_” in this paper.

**Figure 2:**
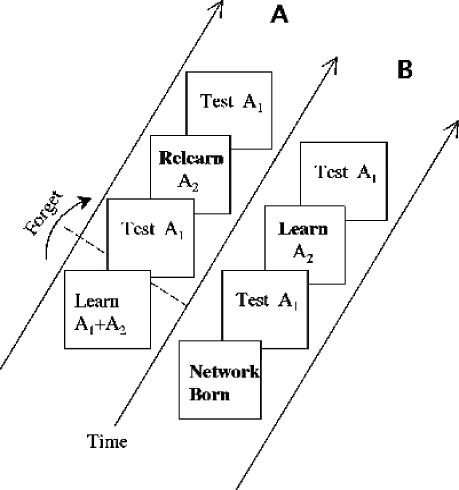
Free-Lunch Learning within and across Generations (A) FLL within the single lifetime of a neural network model. Two subsets of associations *A*_1_ and *A*_2_ are learned. After partial forgetting (see text), performance error on subset *A*_1_ is measured. Subset *A*_2_ is then relearned, and performance error on subset *A*_1_ is measured again. If performance error on *A*_1_ decreases as a result of learning *A*_2_, then FLL has occurred. (B) FLL across generations. Using a genetic algorithm, a network is born with connections similar to those of the network in **a** after it has learned and then partially forgotten subsets *A*_1_ and *A*_2_. Consequently, innate performance error on *A*_1_ is similar to that of the network in **a** after it has partially forgotten both subsets. After measuring performance error on *A*_1_ at birth, subset *A*_2_ is learned for the first time, and performance error on subset *A*_1_ is measured again. After many generations, the innate connection values of each network ensure that if subset *A*_2_ is learned *for the first time,* then this induces automatic learning of subset *A*_1_.

### FLL and Evolution

Now consider an organism *b*_2_ which is born with a genetically specified set of neuronal connections [12]. These connections are organised such that, if *b*_2_ learns one subset *A*_2_ of associations then another subset *A*_1_ is usually learned. In other words, *the organism b*_2_ *happens to be born with neuronal connections similar to the connections of an organism b*_1_ *which had once learned and then forgotten subsets A*_1_ *and A*_2_ (e.g., isotropically distributed around w_0_ in Figure 1). Just as FLL ensures that if organism *b*_1_ relearns *A*_2_ then subset *A*_1_ is usually relearned (see Figure 2), so *if b*_2_ *learns A*_2_ *then A*_1_ *is usually learned*. In both cases, FLL ensures that learning one subset of associations induces learning of the other subset. Critically, whereas the FLL exhibited by organism *b*_1_ depends on previous learning and forgetting, FLL in organism *b*_2_ depends on being born in a state such that the first time *A*_2_ is learned, the associations *A*_1_ are also usually acquired. Such a network can be evolved using a genetic algorithm, as shown below.

The use of two distinct subsets in this paper is clearly unrealistic when considered in the context of skill learning. However, the use of two subsets lies at one extreme along a continuum of tasks. At one extreme, associations are learned one by one in a strict order, and at the other extreme, all associations are learned simultaneously. In a biological context, the components of a skill which are learned first act as “scaffold” for others, and this effectively imposes a temporal order to the acquisition of different skill components. This is the type of scenario assumed for the simulations reported in this paper. Essentially, learning *A*_2_ is assumed to consist of a subset of skill components which provide a scaffold for the skill components in *A*_1_.

### The Geometry of FLL

This section is a brief account of the basic geometry underlying FLL, in the absence of its interactions with evolution. For the present, and without loss of generality (see [10]), we assume that the network has one output unit and two input units, which implies *n* = 2 connection weights, and that *A*_1_ and *A*_2_ each consist of *n*_1_ = *n*_2_ = 1 association, as in Figure 1. Input units are connected to the output unit via weights *w_a_* and *w_b_*, which define a weight vector **w** = (*w_a_*,*w_b_*). Associations *A*_1_ and *A*_2_ consist of different mappings from the input vectors **x**_1_ = (*x*_11_,*x*_12_) and **x**_2_ = (*x*_21_,*x*_22_) to desired output values *d*_1_ and *d*_2_, respectively. If a network is presented with input vectors **x**_1_ and **x**_2_, then its output values are *y*_1_ = **w** · **x**_1_ = *w_a_x*_11_ + *w_b_x*_12_ and *y*_2_ = **w** ·** x**_2_ = *w_a_x*_21_ + *w_b_x*_22_, respectively. More generally, network performance error for *k* associations is defined as

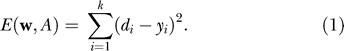



The weight vector **w** defines a point in the (*w_a_*,*w_b_*) plane. For an input vector **x**_1_, there are many different combinations of weight values *w_a_* and *w_b_* that give the desired output *d*_1_. These combinations lie on a straight line *L*_1_, because the network output is a linear weighted sum of input values. A corresponding constraint line *L*_2_ exists for *A*_2_. The intersection of *L*_1_ and *L*_2_ therefore defines the only point **w**_0_ that satisfies both constraints, so that zero error on *A*_1_ and *A*_2_ is obtained if and only if **w** = **w**_0_. Without loss of generality, we define the origin **w**_0_ to be the intersection of *L*_1_ and *L*_2_. A general prerequisite for FLL is that *L*_1_ is not orthogonal to *L*_2_.

We now consider the geometric effect of partial forgetting of both associations, followed by relearning *A*_2_. This geometric account applies to a network with two weights (see Figure 1), and depends on the following observation: if the length of the input vector **x**_1_ is unity, then the performance error *E*(**w**,*A*_1_) = (*d*_1_ − *y*_1_)^2^ of a network with weight vector **w** when tested on association *A*_1_ is equal to the squared distance between **w** and the constraint line *L*_1_ [10]. For example, if **w** is in *L*_1_, then *E*(**w**,*A*_1_) = 0, but as the distance between **w** and *L*_1_ increases so *E*(**w**,*A*_1_) must increase. For the purposes of this geometric account, we assume the length of the input vectors is unity.

If partial forgetting is induced by adding isotropic noise **g** to the weight vector **w** = **w**_0_, then this effectively moves **w** to a randomly chosen point **w**_1_ = **w**_0_ + **g** on the circle *C* of radius *r*, where *r* is the length of **g**, and represents the amount of forgetting. For a network with **w** = **w**_1_, learning *A*_2_ moves **w** to the nearest point **w**_2_ on *L*_1_ [10], so that **w**_2_ is the orthogonal projection of **w** on *L*_2_. Before relearning *A*_2_, the performance error *E*(**w**,*A*_1_) on *A*_1_ is the squared distance *p* ^2^ between **w**_1_ and its orthogonal projection on *L*_1_. After relearning *A*_2_, the performance error *E_post_* is the squared distance *q* ^2^ between **w**_2_ and its orthogonal projection on *L*_1_. The amount of FLL is *δ* = E(**w**_1,_ *A*_1_) − E(**w**_2,_ *A*_1_), and (for a network with two weights) is also given by *Q* = *p* ^2^ − *q* ^2^. The probability *P*(*δ* > 0) of FLL given *L*_1_ and *L*_2_ is equal to the proportion of points on *C* for which *δ* > 0 (or equivalently, for which *Q* > 0). For example, it can be shown that the mean value of this proportion is *P*(*δ* > 0) for a two-weight network like the one shown in Figure 1A. Given the particular configuration shown in Figure 1A, the critical point **w** *_crit_* is defined such that the performance error before and after learning is the same (i.e., *δ* = 0).

### FLL Induces Perfect Performance

Given a network with *n* weights **w** and two subsets *A*_1_ and *A*_2_ of *n*_1_ and *n*_2_ associations, respectively, it is shown that weights **w** ^*^ exist such that learning associations *A*_2_ is guaranteed to yield zero performance error on *A*_1_, provided *n* ≥ *n*_1_ + *n*_2_.

Consider a network with *n* = 2 weights and subsets *A*_1_ and *A*_2_, each of which comprises a single association. Each association defines a constraint line *L*_1_ and *L*_2_, respectively (see Figure 1). If the weight vector **w** is in *L*_1_, then performance error on *A*_1_ is zero, and if **w** is in *L*_2_, then performance error on *A*_2_ is zero. Clearly, if and only if **w** is at the intersection **w**_0_ of *L*_1_ and *L*_2_, then performance error on both *A*_1_ and *A*_2_ is zero. If **w** is not in *L*_2_, then learning *A*_2_ moves **w** from its current position to its orthogonal projection onto *L*_2_ [10]. Crucially, if **w** = **w** ^*^ in Figure 1, then learning *A*_2_ moves **w** to the optimal weight vector **w**_0_. In this case, learning *A*_2_ reduces performance error to zero on both *A*_1_ and *A*_2_, and therefore learning *A*_2_ implies perfect performance on *A*_1_.

This line of reasoning generalises to networks with more than two weights, as follows. If a network has more than two input units, then subsets *A*_1_ and *A*_2_ can have *n*_1_ > 1 and *n*_2_ > 1 associations. If *n* ≥ *n*_1_ + *n*_2_, then *A*_1_ and *A*_2_ define an (*n* − *n*_1_)-dimensional subspace *L*_1_ and an (*n* − *n*_2_)-dimensional subspace *L*_2_, respectively. The intersection *L*_12_ of *L*_1_ and *L*_2_ corresponds to weight vectors which generate zero error on *A* = *A*_1_ ∪ *A*_2_. In this case, the circle in Figure 1 corresponds to an *n*-dimensional hypersphere, with its centre **w**_0_ in *L*_12_. Given that learning *A*_2_ provides an orthogonal projection of **w** onto *L*_2_, and that there exists a **w** = **w** ^*^ such that its orthogonal projection onto *L*_2_ is **w**_0_, it follows that learning *A*_2_ in a network with **w** = **w** ^*^ yields zero performance error on both *A*_2_ and *A*_1_.

Given that the weight vector **w** is genetically specified with finite precision, a network is necessarily born with its weight vector **w** = **w**_1_ at a non-zero distance *r* from the optimal weight vector **w**_0_. This finite precision defines a hypersphere *C* around **w**_0_, and the location of **w**_1_ on *C* determines the amount of FLL. If a network is born with **w**_1_ = **w** ^*^, then learning *A*_2_ induces perfect performance on *A*_1_. If fitness depends on performance on both *A*_1_ and *A*_2_ after learning only *A*_2_, then there is selective pressure for networks to be born with weight vectors close to **w** ^*^, given a specific degree of genetic precision *r*. More generally, there is pressure for networks to be born with weight vectors close to the subspace with contains **w**_0_ and **w** ^*^.

### Terminology: Evolution, Innateness, and FLL

As this paper deals with subtle combinations of evolution and learning, involving two distinct subsets of associations (*A*_1_ and *A*_2_), it is important to be clear about terminology. Specifically, we need to be careful about which subset is being referred to, and whether we are referring to innate performance or not. Accordingly, performance error on *A*_1_ before learning *A*_2_ is called just that, “innate performance error on *A*_1_.” Behaviours that are induced by learning *A*_2_ are called *FLL-induced* behaviours, because they are not innate, nor are they learned, so that performance error on *A*_1_ after learning *A*_2_ is called “FLL-induced performance error on *A*_1_.” If learning *A*_2_ does not affect performance on *A*_1_ (as in condition NoFLL, below), then this is referred to as “post-learning performance.” More generally, performance will be quoted with a specified context (e.g., performance on *A*_1_ after learning *A*_2_).

## Methods

The effect of FLL on evolution was tested by measuring performance on *A*_1_ after learning *A*_2_ across generations. To eliminate the possibility that the observed results are artefacts, the effects of FLL were compared with two control conditions (described below).

Each generation consisted of 1,000 neural networks, each of which consisted of 20 input units and one output unit. The genome of each network was defined by a one-to-one mapping of the *n* = 20 weight values in the network to a single string of *n* genes, where the value of each gene was set to the value of a corresponding network weight. The number of offspring generated by each network was proportional to its fitness, which depended only on its ability to provide the correct desired output value for each of 20, *n*-element input vectors. The mapping from each input vector to its output value defines one association (see Figure 1).

A network's output *y_i_* is a weighted sum of input values 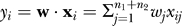 , where *x_ij_* is the *j*th value of the *i*th input vector **x** *_i_*, and each weight *w_i_* is one input–output connection.

The fitness of each network was assessed with respect to its performance error on a single common set *A* = *A*_1_ ∪ *A*_2_ of *m* = 20 associations, where *A*_1_ and *A*_2_ are two disjoint subsets of *n*_1_ = 10 and *n*_2_ = 10 associations, respectively. The *m* associations in *A* were allocated randomly to the two subsets, *A*_1_ and *A*_2_. The subsets *A*_1_ and *A*_2_ were intended to represent different components of a task, and were therefore the same *for all networks,* and *across all generations*. In the first generation, each network's weight values were chosen from a Gaussian distribution (see below for details).

The desired output value *d_i_* for each input vector **x** *_i_* was drawn from a Gaussian distribution with variance 1/*n*. An analytic method was used to solve for the optimal weight vector **w**_0_ which maps inputs to outputs: *d_i_* = **w**_0_ · **x** *_i_* (see below). Given the variances of the inputs and outputs, the expected length of **w**_0_ is unity.

Each new generation was formed from 1,000 matings between 1,000 pairs of networks. The *K* ^th^ network was chosen for mating according to its fitness *F*(*K*) with probability *p*(*K*). Networks were chosen with replacement to ensure that the number of offspring from a given network was proportional to *p*(*K*), which is defined as

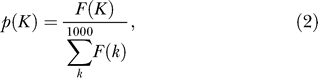
where the denominator ensures ∑*_k_p*(*k*) = 1. Half the weights of each offspring were copied from (randomly chosen) corresponding weight locations in one parent network, and half from the other parent. Aside from mutations, weight values inherited by an offspring were the same as those inherited by its parents (i.e., inheritance was Darwinian, not Lamarckian).

Mutation was applied to each weight with a probability of 0.05, using a uniform probability density function. Then Gaussian noise with a standard deviation of 0.05 was added to the value of those weights that had been chosen for mutation.

There were three conditions: *FLL, NoFLL,* and *NoLearn*, with corresponding fitness functions *F_FLL_, F_NoFLL_,* and *F_NoLearn_*. The initial randomly chosen weight values (see Network Learning Algorithm) of the population of networks were the same in all conditions. Networks were selected for mating according to their performance on the combined set *A* = *A*_1_ ∪ *A*_2_, according to Equation 2 for all fitness functions, as described next.

### Condition FLL

Networks that exhibited high levels of FLL were preferentially selected for mating. Only associations *A*_2_ were learned, but the fitness of each network depended on its performance on both the learned associations *A*_2_ and on the unlearned associations *A*_1_. The fitness *F_FLL_*(*K*) of the *K* ^th^ network is defined in terms of its innate performance error *E_pre_* on *A* = *A*_1_ ∪ *A*_2_, and on its performance error *E_post_* on *A* after learning *A*_2_:

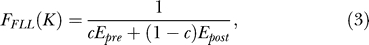
where *E_pre_* and *E_post_* are:

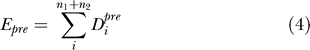


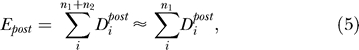
where 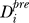 and 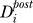 are the network's output errors in response to the *i*th input vector before and after learning *A*_2_ (respectively). The parameter *c* = 0.05 defines the balance between performance error on innate versus post-learning (e.g., FLL-induced) behaviours, and is interpreted as a cost-of-learning parameter (see below). The network's fitness error *D_i_* is a function of the difference *e_i_* = *y_i_* − *d_i_* between the network's response *y_i_* to the *i*th input vector and the desired output value *d_i_*:

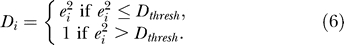
This ensures that output errors above *D_thresh_* have a disproportionately large and detrimental effect on fitness, as shown in Figure 3. This, in turn, ensures that only those networks with “good” performance are likely to be selected for reproduction. The value of *D_thresh_* was set to 0.01.

**Figure 3:**
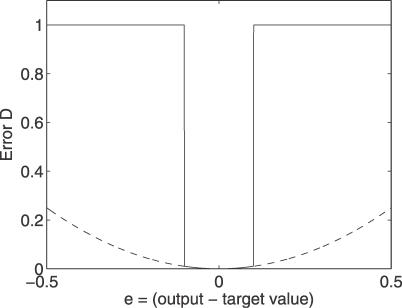
Network Fitness Error Function The response of the network to a given input vector **x** is y = **w** · **x**. Given a desired (target) output *d*, the solid curve shows how the fitness penalty *D* for an incorrect response increases sharply (to unity) if the magnitude of the difference *e* = *y* − *d* is greater than 0.1 (i.e., if *e* ^2^ > 0.01). For comparison, the quadratic error function *e* ^2^ = (*y* − *d*)^2^, which is minimised during learning, is shown as a dashed curve. The range of *e*-values shown are typical for the simulations reported here. See Methods section for details.

For later use, we also define the fitness errors *E_pre_* = *E_pre_*(*A*_1_) + *E_pre_*(*A*_2_), and *E_post_* = *E_post_* (*A*_1_) + *E_post_*(*A*_2_) ≈ *E_post_*(*A*_1_). The approximation here and in Equation 5 emphasises the fact that the total fitness error is attributable almost exclusively to *A*_1_ after learning *A*_2_ (because error on *A*_2_ is then almost zero).

The inclusion of innate performance error *E_pre_* in *F_FLL_* ensures that the cost of learning is taken into account when assessing fitness. If *c* is small, then *E_pre_* tends to be large, so that much learning is required to increase fitness. Conversely, if *c* is large, then *E_pre_* tends to be small, so that little learning is required to increase fitness. Thus, *E_pre_* is an implicit measure of the cost of learning (where *c* multiplies *E_pre_*), and ensures that networks which require minimal learning have high innate fitness (although the small value of *c* used in Equation 3 defines a relatively low cost of learning).

### Condition NoFLL

This was identical to condition FLL, except that the effects of FLL were precluded by making all input vectors in *A* mutually orthogonal, whilst retaining the length of each vector as in condition FLL, using Gram-Schmidt orthogonalisation. This makes the input vectors in *A*_1_ orthogonal to those in *A*_2_, which ensures *L*_1_ and *L*_2_ are orthogonal. This, in turn, ensures that learning *A*_2_ cannot affect performance on *A*_1_. The fitness function *F_NoFLL_* was the same as in condition FLL (i.e., *F_NoFLL_* = *F_FLL_*).

### Condition NoLearn

No learning occurred, so that improvement in performance over successive generations was due only to selection of innate performance on *A*_1_ and *A*_2_. Fitness was defined as *F_NoLearn_* = 1/ *E_pre_*, where *E_pre_* is defined in Equation 4. This is equivalent to setting *c* = 1 in Equation 3.

### Network Learning Algorithm

The network learning algorithm used here involves a type of supervised learning. Note that Equation 1 defines the network error used for learning, whereas Equation 3 defines the fitness of a network.

Each network was initialised with *n* weight values drawn randomly from a Gaussian distribution with unit variance. This was then divided by *n* ^1/2^, which ensures that the expected length of weight vectors in the population is unity.

Given a network with *n* input units and one output unit, the set *A* of *m*, *n*-element input vectors **x** *_i_*: *i* = 1…*m* and *m* desired scalar output target values *d_i_* were chosen randomly from a Gaussian distribution with unit variance. Each input vector was then divided by *n* ^1/2^ so that the expected length of input vectors was unity (i.e., the variance of input values was 1/*n*).

In conditions FLL and NoFLL, each network learned *n*_2_ associations. Rather than using the iterative weight update normally associated with the delta rule, an analytic solution was obtained. Learning *n*_2_ associations consists of finding the orthogonal projection operator which projects the initial weight vector **w**_1_ to its nearest point in the subspace (e.g., *L*_2_) defined by the *n*_2_ input vectors being learned. The end result **w**_2_ is the same as that obtained using the standard delta rule for infinitesimal learning rates [10]. As with the standard delta rule, this yielded a value of approximately zero for post-learning performance error on the learned associations *A*_1_. This type of learning is most plausibly associated with motor learning in the cerebellum and basal ganglia [10].

## Results

The results are based on ten computer simulation runs for each of the three conditions, FLL, NoFLL, and NoLearn, described above, and graphs show the mean of these ten runs. Each run involved a different fixed set *A* of 20 associations. As a reminder, the two free parameters are: 1) the cost-of-learning parameter, which was set to *c* = 0.05, and 2) the threshold of the fitness error function, which was set to *D*_thresh_ = 0.01.

The main results are shown in Figure 4, which is a summary of more detailed results in Figure 5. Condition FLL yields a FLL-induced error (i.e., error on *A*_1_ after learning *A*_2_) of approximately zero after 30 generations, whereas condition NoFLL requires about 60 generations to achieve an error of less than unity. Condition NoLearn (dotted curve) yields the slowest innate learning, and is included for comparison.

**Figure 4:**
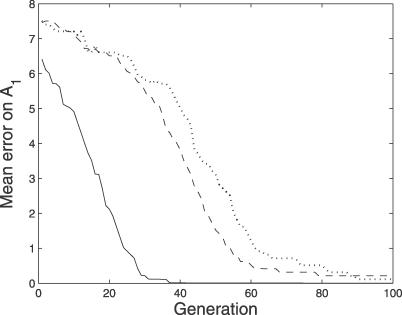
Effect of Free-Lunch Learning on Performance Error The graph shows the mean results of ten computer simulation runs, in three conditions: FLL, NoFLL, and NoLearn (see text). In each run, the fitness of each of 1,000 networks was determined by performance error on a fixed set *A* = *A*_1_ ∪ *A*_2_ of 20 associations; a different set *A* was used for each run. For all graphs in this paper, the median (of error, here) of 1,000 networks was obtained throughout each run, and the mean of ten medians is shown for each generation in each condition (standard errors were no greater than 0.5, and are not shown for clarity). **Condition FLL** (solid line): mean performance error *E_post_*(*A*_1_) on the ten associations in *A*_1_ after learning the ten associations in subset *A*_2_. Learning *A*_2_ had a beneficial effect on performance on *A*_1_ over 100 generations, corresponding to an increase in the amount and prevalence of FLL (see Figure 6). **Condition NoFLL** (dashed line): mean performance error *E_post_*(*A*_1_) was evaluated as in condition FLL, except that the input vectors in *A*_1_ were orthogonal to those in *A*_2_, so that learning *A*_2_ could not have any effect on performance on *A*_1_ (see text). **Condition NoLearn** (dotted line): mean performance error *E_pre_* on *A*_1_ was evaluated “at birth” (i.e., no within-lifetime learning was allowed).

**Figure 5:**
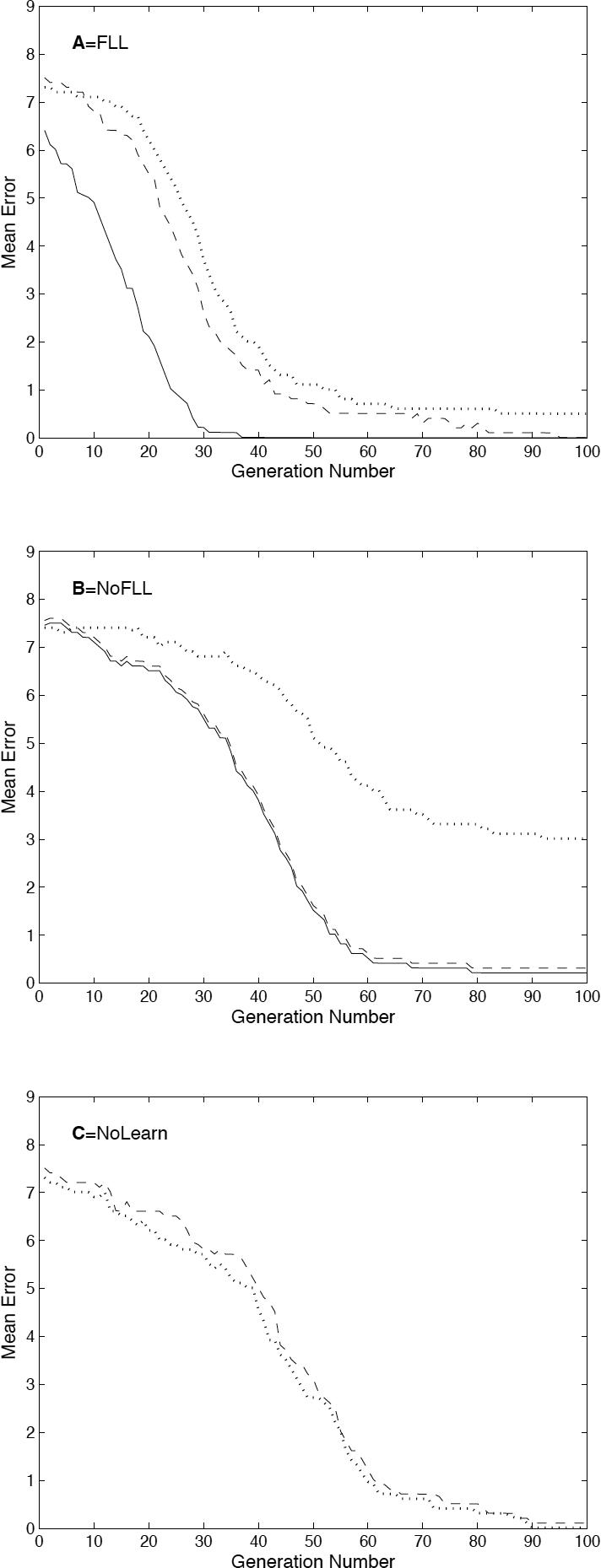
Effect of Free-Lunch Learning on Evolution Performance error in three conditions (FLL, NoFLL, and NoLearn). Different performance errors are drawn as follows: **solid line**, *E_post_*(*A*_1_), performance error on *A*_1_ after learning only *A*_2;_ **dashed line**, *E_pre_*(*A*_1_), innate performance error on *A*_1_; **dotted line**, *E_pre_*(*A*_2_), innate performance error on *A*_2_. (A)** Condition FLL**: mean performance error *E_post_*(*A*_1_) on *A*_1_ (solid line) after learning *A*_2_ decreased over generations most rapidly in this condition. Innate error on *A*_1_ is slightly lower than on *A*_2_. (B)** Condition NoFLL**: precluding FLL ensured that mean innate error on *A*_1_ (dashed line) was essentially the same as after learning *A*_2_ (solid line). The dashed line has been plotted 0.1 units above the solid line for clarity. Innate error on *A*_2_ is large because the fitness cost of innate errors is low (*c* = 0.05). (C)** Condition NoLearn**: mean innate performance error *E_pre_*(*A*_1_) and *E_pre_*(*A*_2_) on *A*_1_ and *A*_2_. For comparison, mean performance errors on *A*_1_ in each condition (i.e., the solid lines here) are summarised in Figure 4.

**Figure 6:**
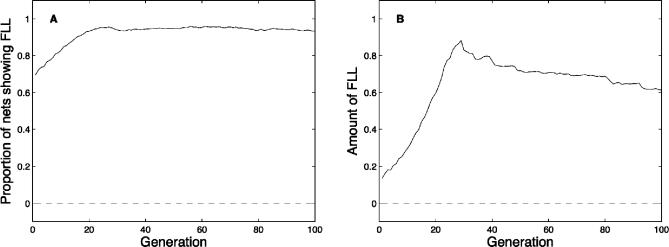
Prevalence and Amount of Free-Lunch Learning The lines in each graph correspond to the conditions: FLL = **solid line**, NoFLL = **dashed line**. Performance error on *A*_1_ was tested after learning *A*_2_ in conditions FLL and NoFLL. Each plotted line is the mean of ten computer simulation runs (see Figure 4 for details). (A)** Prevalence of FLL**: the proportion of networks which showed improved performance on *A*_1_ after learning *A*_2_. In condition FLL, the prevalence of FLL was non-zero in the first generation, as expected (see text), and increased across subsequent generations. In condition NoFLL, the prevalence remained at zero, as expected. (B)** Amount of FLL**: in condition FLL, the amount of FLL increased dramatically over the first 30 generations. The absence of FLL in condition NoFLL is as expected (see text). The amount of FLL defined for this graph only is the difference in fitness error on *A*_1_ before and after learning *A*_2_, expressed as a proportion of the error on *A*_1_ before learning *A*_2_; or, equivalently, as [*E_pre_*(*A*_1_) − *E_post_*(*A*_1_)]/*E_pre_*(*A*_1_).

The proportion of networks that exhibit FLL over generations is shown in Figure 6A, and the amount of FLL is shown in Figure 6B. The proportion and amount of FLL increases in condition FLL, as indicated by the solid line in each figure. The zero prevalence of FLL in condition NoFLL (dashed line) is associated with zero FLL as indicated in Figure 6B. More detailed results are shown in Figure 5A–5C.

### Performance on *A*_1_

Performance on *A*_1_ (solid line in Figure 5A) after learning *A*_2_ is better in condition FLL than in condition NoFLL (solid line, 5B). Innate performance on *A*_1_ is also better in condition FLL (dashed line, Figure 5A) than in conditions NoFLL (dashed line, Figure 5B) and NoLearn (dashed line, Figure 5C). Together, these results suggest that FLL accelerates both the rate at which FLL-induced behaviours (*A*_1_) appear, as well as the rate at which FLL-induced behaviours (*A*_1_) become genetically assimilated.

### Performance on *A*_2_

Innate performance on subset *A*_2_ is better in condition FLL (dotted curve, Figure 5A) than in conditions NoFLL (dotted curve, Figure 5B) and NoLearn (dotted curve, Figure 5C).

Learning *A*_2_ reduces error on subset *A*_1_ even in the first generation in conditions FLL (Figure 5A). Additionally, the proportion of networks showing FLL is greater than 0.5 (Figure 6A), and the amount of FLL is greater than zero (Figure 6B) in the first generation. These effects are not due to any special properties of the networks nor of the associations. Indeed they are entirely expected, and are consistent with the theoretical analysis in [10]. In essence, FLL is observed in the first generation because it is very unlikely that the mainfolds *L*_1_ and *L*_2_ defined by *A*_1_ and *A*_2_ (respectively) are orthogonal, so that learning *A*_2_ usually reduces error on *A*_1_ (albeit by a small amount in the first generation).

## Discussion

Before discussing results in detail, it is important to clarify precisely what is being claimed here. The main claim is that, given a population of organisms which can learn, the presence of FLL accelerates the rate at which a given set of advantageous behaviours evolves relative to populations which 1) can learn but which do not have FLL (e.g., condition NoFLL) and 2) cannot learn (e.g., condition NoLearn). Specifically, it is claimed that FLL accelerates the appearance of adaptive behaviour, both in its innate form and as FLL-induced behaviour, and that FLL can accelerate the rate at which learned behaviours become innate.

It is also claimed that FLL increases the rate at which a set of behaviours (e.g., *A*) is acquired within a lifetime. Clearly, if learning one subset (e.g., *A*_2_) induces learning of another subset (e.g., *A*_1_), then the amount of learning required to learn both subsets (e.g., *A*) is reduced.

It is worth noting that FLL is not related to generalisation (see [10]), which cannot therefore be responsible for the effects reported here.

### Task Difficulty

The task was purposely made difficult, such that network outputs which were not close to desired target values were assigned an error value of unity. This heavily penalises networks that do not generate near-correct responses. This type of task may emulate tasks for which being “almost correct” provides no fitness benefit. Such tasks are exemplified by a predator which almost catches prey (e.g., a kingfisher almost catching a fish, or where each failed attempt yields a large fitness cost), or where learning is incremental and stepwise (e.g., learning to catch progressively larger prey). Such tasks give rise to “needle-in-a-haystack” search spaces [5], which have rugged or uncorrelated landscapes [13].

### Is Accelerated Evolution due to Learning?

A cogent critique of research by Nolfi et al. [14] argues that accelerated evolution (specifically, assimilation) is a generic consequence of learning per se [15]. In results not shown here, replacing *A*_2_ with a new, randomly chosen subset every generation in condition FLL yields a more gradual evolution of FLL-induced and innate behaviours than is obtained in any of the conditions used here. This effectively excludes the possibility that the accelerated evolution reported here is due to learning per se.

### Reaction Norms

In terms of evolutionary theory, FLL-induced behaviours can be considered as the establishment of a new reaction norm. The specific “environment” that induces the reaction norm is learning a particular subset of behaviours (*A*_2_), and the phenotypic reaction to this environment is another subset of behaviours (*A*_1_).

### Genetic Assimilation

FLL does not necessarily force FLL-induced behaviours to become genetically assimilated. In fact, there is a tradeoff between the amount of acceleration induced by FLL and the extent to which behaviours become innate. If the cost-of-learning parameter is set to *c* = 1, then there is no incentive for FLL to increase over generations. In contrast, if *c* ≈ 0 (as in the simulations reported here), then the rapid evolution of FLL-induced behaviour shown in Figure 5A (solid line) is obtained, alongside the slower evolution of innate behaviour (dashed line in Figure 5A). Thus, even the small value of *c* (0.05) used here puts pressure on learned behaviours to become innate, as indicated by the decreasing innate performance errors on *A*_1_ (dashed line) and *A*_2_ (dotted line) in Figure 5A.

In practice, learning always has a non-zero fitness cost, if only in terms of the time required for that learning to occur. This is because time spent learning is time spent not eating, or time spent being eaten, both of which reduce fitness. Thus, the small value of *c* used here represents one value along the spectrum of learning costs. It therefore seems likely that even the simplest learned behaviours have a tendency to become innate, and that this tendency increases with the cost of learning. For example, in results not shown here, increasing the cost-of-learning parameter *c* decreases the rate at which FLL-induced performance on *A*_1_ improves, and increases the rate at which performance on *A*_1_ and *A*_2_ becomes innate (innate performance with *c* = 1 is effectively obtained in condition NoLearn (see Figure 5C)). It is therefore not easy to classify the effect reported here as a clear-cut example of the Baldwin effect [6], although these effects are almost certainly related.

### General Free-Lunch Effects

The basic geometry which underpins FLL within a lifetime (as in [10]) and across lifetimes (as here) can also be applied in two other contexts: 1) evolution of innate behaviours without learning, and 2) evolution of general phenotypic traits. These two cases are considered in the next two paragraphs. Both of these effects require the presence of environmental conditions that fluctuate over successive generations (e.g., fluctuations in temperature induced by ice ages, salinity, prey numbers, or predation pressure).

1) Accelerated evolution of innate behaviours without learning can be understood by considering an organism that has no learning ability, and which relies on genetic specification of its neuronal connections [12]. Natural selection ensures that its neuronal connections at birth yield innate behaviour matched to its environment. If the environment changes, then natural selection will induce a corresponding shift to a new set of innate connections. If the environment then shifts back to its original state, then organisms' connections will tend to revert to their original values. Let us assume that some connections revert faster than others over successive generations. For example, some connections may be specified by genes linked to other innate behaviours, and this genetic linkage would tend to reduce the rate of genetic change. In fact, for simplicity, assume that half of the connections revert quickly and half revert slowly. If the required behaviours are encoded as distributed representations, then this connection reversion will induce a FLL-type effect, such that *all* associations benefit from the reversion of a proportion (half here) of connection values.

2) Accelerated evolution of general phenotypic traits can be understood if we assume an extreme form of pleiotropy: that each of a given set of genes affects every phenotypic trait. This is equivalent to assuming that the genome is a *distributed representation* of the phenotype. Consider a population in which the fittest organism has a genome **w**_0_ which is perfectly adapted to its environment *e*_0_. If the environment changes to *e*_1_, then the fittest organism's genome will eventually evolve to a new state **w**_1_ that is suited to *e*_1_ (this is analogous to forgetting in FLL). Now, consider what happens if the environment changes back to *e*_0_. The fittest organism's genome will be forced back toward **w**_0_, but inevitably some genes will revert faster than others. For the sake of argument, assume that a subset *G*_2_ of genes revert to their original values, while others *G*_1_ remain as they were in **w**_1_ (this is analogous to relearning only *A*_2_). Because each gene in *G*_2_ contributes to every phenotypic trait, the reversion of genes in *G*_2_ to their original values will push the entire phenotype back toward its state in the original environment *e*_0_. Thus, the reappearance of an entire set of phenotypic traits (e.g., changes in size) can occur more quickly if those traits are encoded within a set of pleiotropic genes than if each trait is represented by a non-pleiotropic gene, and suggests a form of *free-lunch evolution*.

### Conclusion

It has been demonstrated that FLL accelerates the evolution of behaviours in neural network models. Given that FLL appears to be a fundamental property of distributed representations, and given the reliance of neuronal systems on distributed representations, FLL-induced behaviours may constitute a significant component of apparently innate behaviours (e.g., nest-building). Results presented here suggest that any organism that did not take advantage of such a fundamental and ubiquitous effect would be at a selective disadvantage. Finally, if FLL accelerates evolution in the natural world, then it may have been involved in the Cambrian explosion, an explosion that began when brains (and therefore learning) first appeared.

## Abbreviations

FLL: free-lunch learning
